# Ecosystem Service Valuations of Mangrove Ecosystems to Inform Decision Making and Future Valuation Exercises

**DOI:** 10.1371/journal.pone.0107706

**Published:** 2014-09-22

**Authors:** Nibedita Mukherjee, William J. Sutherland, Lynn Dicks, Jean Hugé, Nico Koedam, Farid Dahdouh-Guebas

**Affiliations:** 1 Laboratory of Systems Ecology and Resource Management, Université Libre de Bruxelles, Brussels, Belgium; 2 Laboratory of Plant Biology and Nature Management, Vrije Universiteit Brussel, Brussels, Belgium; 3 Conservation Science Group, Department of Zoology, University of Cambridge, Cambridge, England, United Kingdom; 4 Centre for Sustainable Development, Ghent University, Gent, Belgium; Texas A&M University at Galveston, United States of America

## Abstract

The valuation of ecosystem services is a complex process as it includes several dimensions (ecological, socio-cultural and economic) and not all of these can be quantified in monetary units. The aim of this paper is to conduct an ecosystem services valuation study for mangroves ecosystems, the results of which can be used to inform governance and management of mangroves. We used an expert-based participatory approach (the Delphi technique) to identify, categorize and rank the various ecosystem services provided by mangrove ecosystems at a global scale. Subsequently we looked for evidence in the existing ecosystem services literature for monetary valuations of these ecosystem service categories throughout the biogeographic distribution of mangroves. We then compared the relative ranking of ecosystem service categories between the monetary valuations and the expert based analysis. The experts identified 16 ecosystem service categories, six of which are not adequately represented in the literature. There was no significant correlation between the expert based valuation (the Delphi technique) and the economic valuation, indicating that the scope of valuation of ecosystem services needs to be broadened. Acknowledging this diversity in different valuation approaches, and developing methodological frameworks that foster the pluralism of values in ecosystem services research, are crucial for maintaining the credibility of ecosystem services valuation. To conclude, we use the findings of our dual approach to valuation to make recommendations on how to assess and manage the ecosystem services provided by mangrove ecosystems.

## Introduction

The sustainable provision of the goods and services that we derive from nature (i.e. ecosystem services) is essential to human well-being and survival [1]–[3]. The consequences of the widespread decline of these ecosystem services (ES) have been amply demonstrated by research in the past decade [4], [5]. Over half (approx. 60%) of the major global ES have either been degraded or used unsustainably [2]. These ES range from provisioning services such as freshwater and fisheries, to regulating services such as air and water purification and climate regulation, to cultural and aesthetic services. For example, two-thirds of the world population is projected to be under water stress by 2025 [6] while one third of the world's major fisheries had already collapsed by 2003 and many continue to decline [7], [8].

One of the steps in addressing this situation may be through valuation of the critical ES particularly in monetary terms. There is a widespread notion that valuation exercises might help decision makers appreciate the value of ES to society and the anticipated cost of their imminent loss [9], [10]. Economic valuation in particular is often expected to be a useful tool to support conservation policy decisions and governance [10], [11]. Following the seminal work by Costanza et al. [3] and the Millennium Ecosystem Assessment [2] which involved 1300 scientists, valuation of ES has received unprecedented attention in the last decade [11]–[13]. A range of different valuation methods have been designed and new networks have been formed for better exchange of information in this rapidly evolving field [14], [15]. In a recent study, Costanza et al. [16] state that valuing ecosystems and their services is inevitable (even if it is implicit) in any decision involving trade-offs concerning them. Economic valuations of ES improve the transparency of the valuation process and may thereby usher in better decision making about ES [16].

Until now, valuation methods, data and classification systems for ecosystems were developed predominantly for terrestrial ecosystems while coastal ecosystems have received scant attention [17], [18]. Peer-reviewed literature on global economic valuations of coastal forests like mangrove ecosystems is rather limited [19], [20]. Mangrove ecosystems are tidally influenced wetland forests present in 123 countries [21]. Some worldwide assessments have considered mangroves as a subset of other coastal ecosystems in the economic evaluations of ES. However, the contribution of mangrove ecosystems to the aggregate economic value is often hard to disentangle. The possible pitfall in such large-scale studies is that there is considerable overlap with several other ecosystem types, possibly leading to double counting. For instance, mangroves are either combined with tidal marshes (wetlands) in Costanza et al. [16] or divided into ‘tropical forests’, ‘coastal systems’ and ‘coastal wetlands’ in de Groot et al. [15].

Mangrove ecosystems merit further attention in their own right. The number of people living within 10 km of significant mangrove areas might rise to 120 million by 2015 [22]. The bulk of this population resides in developing countries in Asia and West and Central Africa and is significantly dependent on mangrove resources for daily sustenance and livelihood. In coastal regions dominated by sandy beaches where timber species are scarce, mangrove plants are often the only available source of fuelwood and timber for construction of houses in tropical developing countries [23]. The linkages between mangroves and fisheries have been documented in ecological literature [23]–[26]. Even though the absolute economic value (monetary value) of the resources may not be high [27] (e.g. some species of snails and crabs have no market value but they are consumed when no other food or protein source is available), the relevance of these biological resources may be paramount for the communities dependent on them. Mangroves in such cases may be considered human life-support systems. Mangrove forests are also important for their role in providing coastal protection against recurrent storms and other natural hazards [28]. The dense network of roots bind the soil and trap the sediment and suspended particulate matter in deltaic settings [29]. Mangroves are also known to be the most carbon rich forests in the tropics, reported to have 1023 Mg C per hectare of forest including soil carbon [30]. Per unit area, this is higher than any other marine ecosystem, such as seagrass beds and salt marshes. In spite of their socioeconomic importance, mangrove area has declined by 30–50% in the past 50 years, a rate higher than most other biomes [31]. Remnant mangroves are severely threatened, with up to 40% of the mangrove plant species being susceptible to extinction in some regions [32]. This loss and degradation may seriously undermine the ability of mangroves to provide valuable ES for present and future generations [33]. Stemming this loss is urgent and requires better management, and restoration of, intact and damaged mangrove ecosystems. It also calls for systematic assessments of current ‘stocks’ and ‘flows’ of ES to ensure the sustainable use of these resources [11]. Since mangroves have not received their due share of conservation attention and the rate of decline has not been curbed, the issue of giving incentives after establishing monetary value of ES becomes more important.

To date, there are only three large-scale economic assessments specifically targeted towards mangrove ecosystems [19], [20], [34]. While Vo et al. [34] provided a review of the methods used for valuation of mangrove ES, Brander et al. [20] focused only on mangroves in South East Asia. Salem and Mercer [19] on the other hand, looked at economic valuation of mangroves globally. None of these studies had attempted to bridge over to management implications of the ES values or identify the gaps in the current valuations of mangrove ES. All the above mentioned studies were conducted by economists, and were based on aggregation of case studies of economic valuations of mangrove ES in different geographic areas. They are thereby limited to only those estimates available in the existing literature and do not go beyond them. Since a large proportion of the ES derived from mangroves may lie in the ‘grey market’ (i.e. may not have a direct market price), it may be difficult for economists to identify and value the full range of nature-human interlinkages that involve mangroves especially in the case of developing countries. Martín-López et al. [35] emphasize the importance of a multidimensional valuation of ecosystem services, including their non-monetary values.

Combining monetary assessments with valuation by expert based knowledge is one such approach, which may provide interesting insights that are otherwise not possible to attain through conventional monetary valuations alone. The expert based Delphi technique is particularly useful in this context and has been found to be useful in other ecosystems [36], [37]. Since most ES are heavily reliant on the proper functioning of the ecosystems [38], integrating ecological knowledge from experts in the relevant field can be considered as an important first step in the mangrove valuation exercise. Though some economists recognize that functionality of ecosystems per hectare has been declining in several cases [16] and this in turn affects the supply of ES, rarely has this aspect been given its due attention in economic assessments except in a few cases [39]. The current study proposes a slightly different framework that turns the valuation process on its head by beginning primarily with ecologists rather than economists.

The aim of this paper is to identify the gaps in current economic valuations of mangrove ES and to suggest ways to inform decision making for better management of mangrove resources. We used an inclusive approach to value ES of mangroves that could then be used to inform governance and decision-making. We used an expert-based participatory approach to identify, categorize and rank the various mangrove ES categories. Hence this study presents new information beyond traditional meta-analysis [19] or systematic reviews of existing economic valuations, and thereby captures the ES that have not yet been economically valued. Further on, we searched for evidence in the existing ES literature for monetary valuations of these ranked ES categories. We compared the relative ranking of ES categories by the monetary valuations and by the expert based knowledge. In order to make the expert based ES categories comparable to other ES valuations, they were collated according to the Common International Classification of Ecosystem Services (CICES) v4.3 (http://cices.eu/). This allowed us to make practical suggestions at the level of management. Lastly, we discuss the possible future trajectories of the valuation and management options for the different ES categories.

## Methods

### 1. The Delphi technique

#### 1.1. Brief description of the method

The Delphi technique is defined as ‘a method for structuring a group communication process so that the process is effective in allowing a group of individuals as a whole to deal with a complex problem’ [40]. In this technique, expert judgement is elicited in an iterative, anonymous survey with feedback to the participants between each round. The Delphi technique allows all the participants to evaluate the information produced by the group and weigh dissenting views and the consensus is expected to increase from round to round. Individual participants may reconsider or explain their suggestions based upon their evaluation of new information provided. Essentially, the Delphi technique transforms diverse individual knowledge to create a collective wisdom without the domination of individual views [41]–[43].

#### 1.2. Mangrove Delphi technique

We invited 106 mangrove experts (scientists, reserve managers and field-based conservationists) to participate in the survey. The current work is part of a larger global survey on biodiversity and ecosystem functioning of mangroves [44]. The criteria used for selection and invitation of experts have been explained in detail in [44] and have also been included in the material S1. Briefly, the experts consisted of established mangrove ecologists, mangrove managers and on-ground restoration biologists who were/are involved in mangrove research and management for at least 8 years. Care was taken to select experts outside our research group.

**Figure 1:**
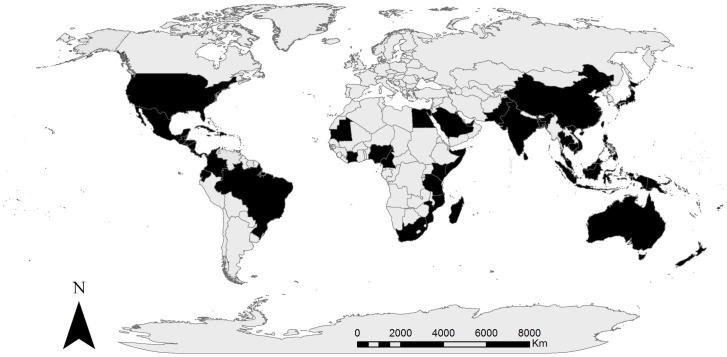
Map representing countries (coloured black) where the experts have conducted primary research.

In the first round of the survey, thirty-five experts participated (34% of those invited), while nineteen experts participated in the second round (54% of the first round participants). Respondents of the first round (n =  thirty-five) had carried out field research on mangroves in fifty-five countries (Fig. 1). The respondents who completed the entire survey (both rounds) had published 691 peer-reviewed co-authored publications on mangroves and had been cited over 10,829 times (without self-citations). The respondents had a median of 20 years of experience in mangroves and their cumulative expertise covers all mangrove species and environments based on spatial outline in [21].

#### 1.3 Procedure of the survey

The Delphi technique survey consisted of two rounds, conducted within a time-frame of four months (5th November, 2011 to 5th March, 2012). The entire survey was conducted online and a website was designed specifically for this survey [45]. The online survey questionnaires in both rounds were designed using Google Forms.

The survey consisted of five steps. (i) The first round of questions of the Delphi technique was prepared and an online invitation for participation was sent to the selected 106 experts. Typical of the Delphi technique process, the first round questions were open-ended and the experts were asked to suggest the various ES provided by mangrove ecosystems based on their field experience. In order to avoid the confusion between ecosystem functions (defined by Reiss et al. [46] as the changes in energy and matter over time and space through biological activity) and ecosystem services (defined as products of ecosystem functioning that are of (usually socioeconomic) value to humans by [46], only the latter has been dealt with in this study. The question on ecosystem functions has already been addressed in [44]. (ii) The respondents completed the survey and sent it back to us. (iii) The responses were analyzed and collated into a list of 16 ES categories based on their similarity. A feedback report was prepared and uploaded on the website. (iv) In the second round, the experts scored these 16 categories of ES that they had suggested in the previous round. The experts were asked to score options on a Likert scale of 1–5, where “1” indicated low value and “5” indicated high value [47]. Later, to rank the categories, the Likert scale scores for each ES category were given corresponding weights (e.g.Likert score 1 =  weight 1, Likert score 5 =  weight 5) and multiplied by the number of votes for that option to generate a total weighted score for that ES. Further on, these weighted scores were converted to a percentage scale to generate a ranking of the ES categories. Since the experts who participated in the first round were requested to participate in the second round, the participants were self-selected in the second round, contained within the first selection. (v) Thereafter, the second round responses were analysed, compiled into a feedback report and uploaded on the website. Based on the categorization of von der Gracht [48] for consensus measurement, we followed a ‘subjective analysis’ approach. It was felt that a third round would not add to the understanding provided by the first two rounds. Thus, the Delphi technique was terminated after the second round. Unless otherwise stated, the results from the second round of the Delphi technique are presented here.

We did not obtain ethics approval for this exercise as only those experts who were willing to give their views took part in the survey. The respondents were given the choice of being acknowledged or remaining anonymous at the end of the second round of the survey. Names of those mangrove experts who wished to be acknowledged can be found in the acknowledgements section. In addition, the survey was anonymous while it was being conducted, similar to other studies in the literature [49].

### 2. Estimates of economic value

Existing global databases like the Ecosystem Services Valuation Database [50] formed a valuable starting point for the data collation. The Economics of Ecosystems and Biodiversity (TEEB) database covers more than 1310 values of ES for a range of different ecosystems from 267 references. We selected only those values from these case studies that specifically belonged to mangroves. The bibliometric search was performed using the keywords (“mangrove” or “mangroves”) and (“ecosystem service” or “economic valuation” or “value”) in a search query within the ISI Web of Science database (http://apps.webofknowledge.com), Google and Google Scholar from 1955–2013 (as of 14th January, 2014). After an initial screening of over 6000+ records, only those studies in peer reviewed literature were retained that specifically mentioned (a) a monetary value for the ecosystem service in USD, (b) the valuation method, (c) the location (country) where the study was conducted. Datasets from published reviews (peer-reviewed) were also taken into consideration [15], [19]. We do acknowledge that economic valuations of mangrove ES published in ‘grey literature’ may exist, but it was beyond the scope of this paper to include them. In addition, documents published in any language other than English have not been covered in this study.

All values which were published after [15] were standardized to 2007 estimates based on the procedure described in detail in the TEEB database [50] to maintain parity. Briefly, all economic estimates in the original case studies were converted into the official local currency. Then these values were adjusted to 2007 values and finally they were converted to international dollars using the purchase power parity (PPP) conversion factor (‘local currency per international $’ series). The official exchange rates, GDP deflators and PPP conversion factors from the World Bank World Development Indicators 2009 were used to standardize values estimated in different years and different currencies.

Mathematically, 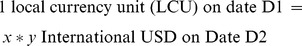
(where, *x* =  deflation of LCU between D1 and D2, *y* =  PPP forex rate in USD per LCU on D2).

Since estimates of the sample size and variance of the original studies were absent in most of the original estimates it was difficult to conduct a meta-analysis following the guidelines set by Vetter et al. [51].

Values obtained from the various sources (n =  thirty four) were collated according to the Common International Classification of Ecosystem Services (CICES) classification system (http://cices.eu/) and the response categories of the Delphi technique (see previous section on the Delphi technique). Average values for each category were calculated. These average economic values were sorted to produce a ranking of the ES categories. The correlation between the ranking produced by the economic valuations and by the experts in the Delphi technique was tested using the Spearman's Rank correlation calculated in the statistical programme ‘R’. In case a category suggested by the Delphi technique fell across two or more categories in the CICES framework, the economic values were aggregated to the Delphi ES category for comparison.

## Results

### 1. Expert based valuation

**Figure 2:**
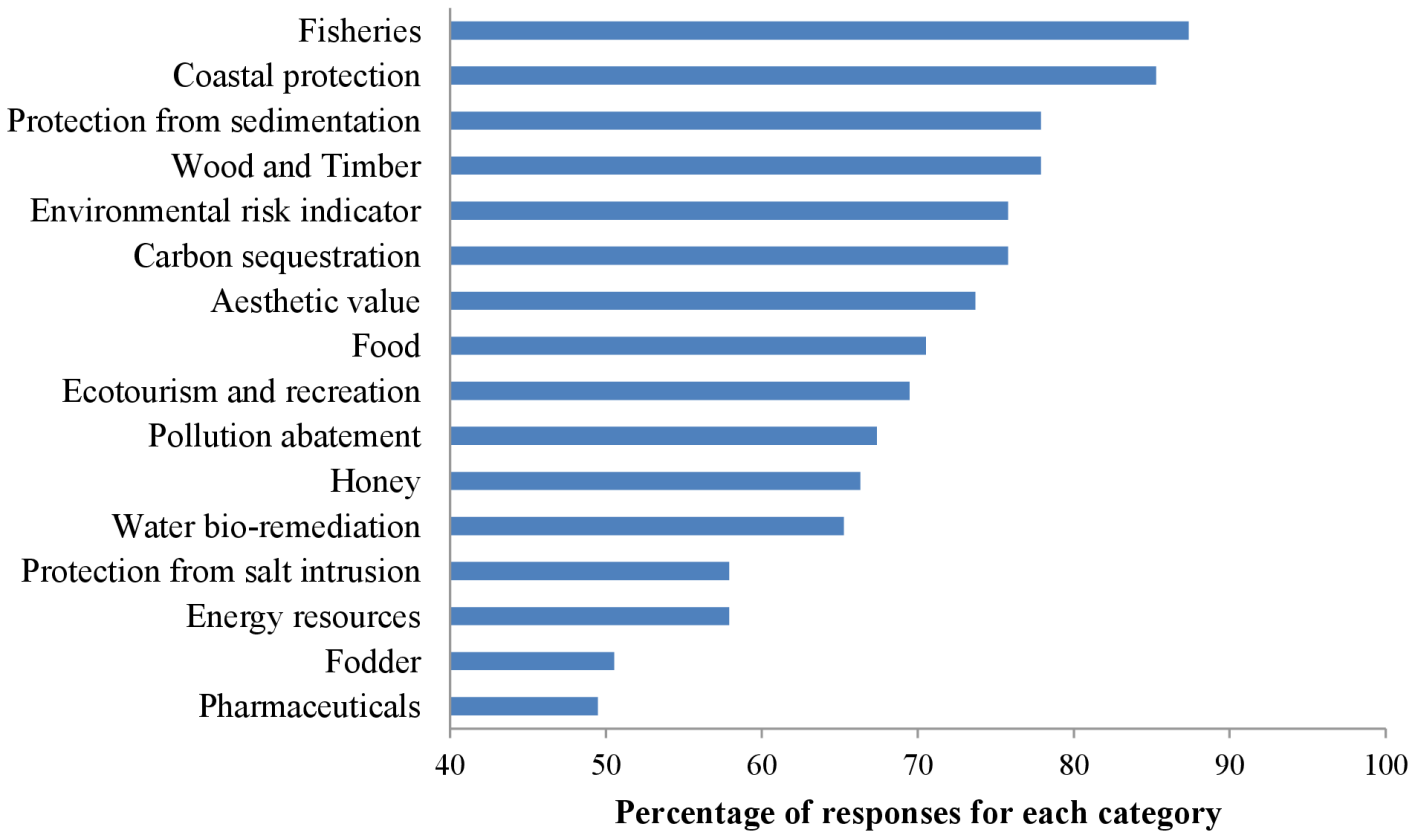
Ranking of the ecosystem service categories of mangroves based on the scores given by the experts in the Delphi technique.

There was a high level of consensus amongst the experts (as indicated by the stability of responses after only two rounds of the Delphi technique) even though they worked in a range of different field sites, biogeographical regions and socio-economic settings across the globe. The 16 ES categories identified by the expert panel are shown in Table 1 based on the CICES framework. The role of mangroves in fisheries, coastal protection, protection from sedimentation and provisioning for wood and timber were identified to be the top three ES of mangrove ecosystems (Fig 2). Three of these ES fall under the category of regulation and maintenance services according to CICES, with “fisheries” being spread over both provisioning (nutrition) and regulation and maintenance (nursery function). Mangrove ecosystems were also identified to be important environmental risk indicators and carbon sequesters. In the context of climate change, the emphasis on coastal protection and protection from sedimentation are particularly important, given the location of mangroves close to the coast and the rapid decline of mangrove area [52] in the past few decades.

**Table 1:** Ecosystem service categories identified by the mangrove Delphi, grouped according to the Common International Classification of Ecosystem Services (CICES) v4.3 (http://cices.eu/).

CICES for ecosystem accounting					
Section	Division	Group	Class	Class type	Delphi technique categories
**Provisioning**	Nutrition	Biomass	Wild animals and their outputs	Animals by amount, type	Fisheries (food)
					Honey
			Animals from *in-situ* aquaculture	Animals by amount, type	Fisheries (aquaculture)
	Materials	Biomass	Fibres and other materials from plants, algae and animals for direct use or processing	Material by amount, type, use, media (land, soil, freshwater, marine)	Wood and timber
					Pharmaceuticals
			Materials from plants, algae and animals for agricultural use		Fodder
	Energy	Biomass-based energy sources	Plant-based resources	By amount, type, source	Energy resources
**Regulation & Maintenance**	Mediation of waste, toxics and other nuisances	Mediation by biota	Bio-remediation by micro-organisms, algae, plants, and animals	By amount, type, use, media (land, soil, freshwater, marine)	Water bio-remediation
		Mediation by ecosystems	Filtration/sequestration/storage/accumulation by ecosystems	By amount, type, use, media (land, soil, freshwater, marine)	Pollution abatement, Environmental risk Indicator
	Mediation of flows	Mass flows	Mass stabilization and control of erosion rates	By reduction in risk, area protected	Protection from sedimentation
			Buffering and attenuation of mass flows		Protection from salt intrusion
		Gaseous/air flows	Storm protection	By reduction in risk, area protected	Coastal protection
	Maintenance of physical, chemical, biological conditions	Lifecycle maintenance, habitat and gene pool protection	Maintaining nursery populations and habitats	By amount and source	Fisheries (nursery)
		Soil formation and composition	Decomposition and f ixing processes		Carbon sequestration
		Atmospheric composition and climate regulation	Global climate regulation by reduction of greenhouse gas concentrations	By amount, concentration or climatic parameter	Carbon sequestration
**Cultural**	Physical and intellectual interactions with biota, ecosystems, and land-/seascapes (environmental settings)	Intellectual and representative interactions	Entertainment		Ecotourism and recreation
			Aesthetic		Aesthetic value

The first five columns belong to the CICES framework and the results of the Delphi technique are included in the last column.

### 2. Economic valuation

**Figure 3:**
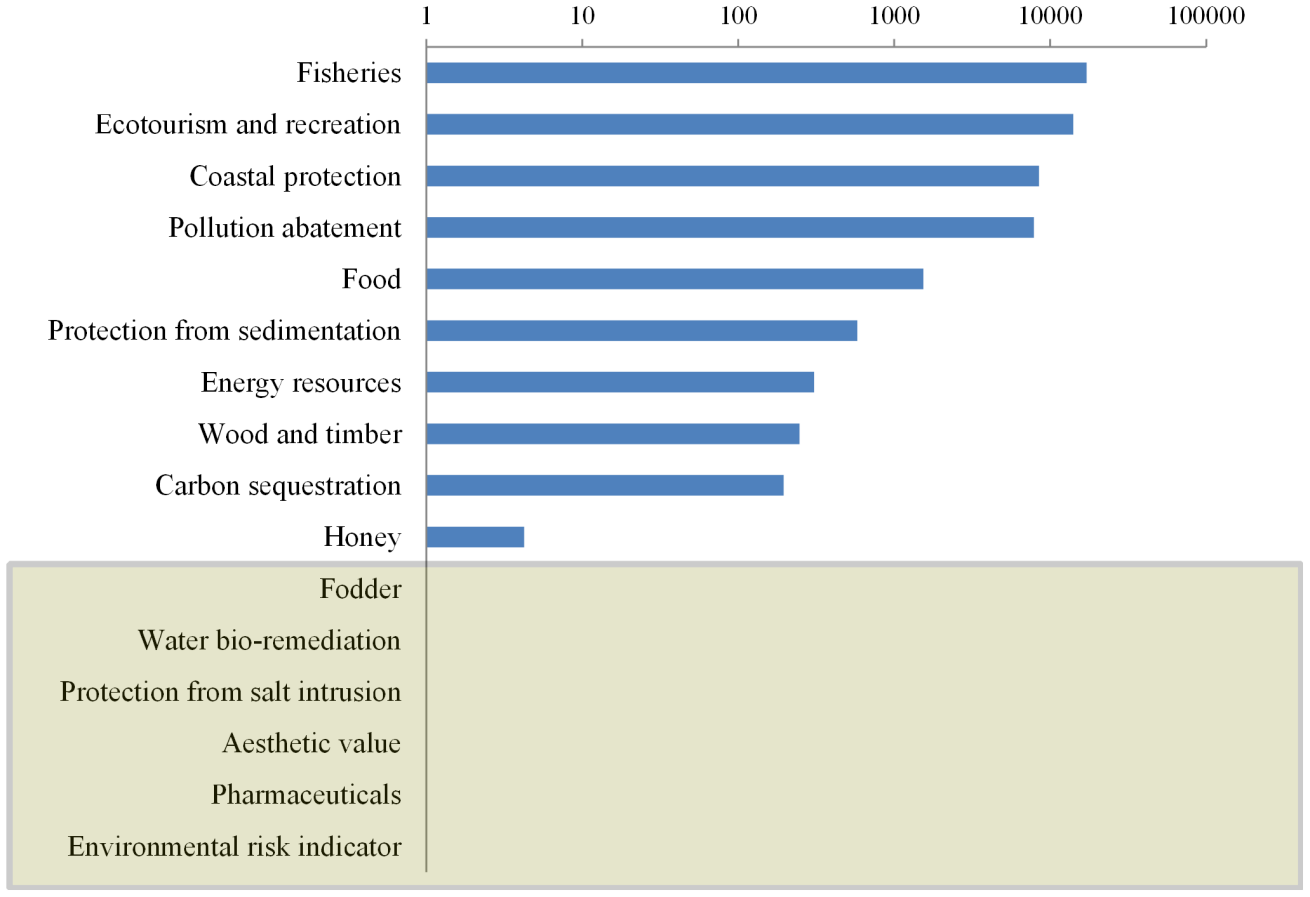
Average economic values (log scale) of the different ecosystem services noted in literature standardized for 2007 Int$/ha*yr. The five ecosystem services for which no economic values were found, are highlighted in the shaded box.

The economic values of the mangrove ES standardized for the 2007 international dollar is given in Table 2. According to the existing peer-reviewed literature, ecotourism and fisheries generated the highest economic value (including subsistence) based on the 2007 estimates (Fig 3). It should be noted here that the role of mangroves in fisheries has been split into three categories based on the CICES classification but their combined value is presented in Table 2. Coastal protection also ranked highly (third) according to the average estimates of economic values.

**Table 2:** Ecosystem services provided by mangroves sorted according to their mean economic values (2007 Int$/ha*yr).

Delphi technique categories	Mean economic value (2007 Int$/ha*yr)	No. of estimates	Economic rank	Delphi technique rank
Fisheries (nursery and aquaculture)	17090.1	25	1	1
Ecotourism and recreation	14072.14	10	2	7
Coastal protection	8459.12	9	3	2
Pollution abatement	7859.92	2	4	8
Food	1535.21	16	6	6
Protection from sedimentation	579.28	1	7	3
Energy resources	306.92	8	8	12
Wood and timber	247.34	3	9	3
Carbon sequestration	195.23	3	10	4
Honey	4.23	2	11	9
***Fodder***	0	0	0	13
***Water bio-remediation***	0	0	0	10
***Protection from salt intrusion***	0	0	0	11
***Aesthetic value***	0	0	0	5
***Pharmaceuticals***	0	0	0	14
***Environmental risk indicator***	0	0	0	4

Six of the 16 ecosystem services (highlighted in bold and italics) identified by experts do not have adequate valuations in the peer-reviewed ecological economic literature.

Six of the ES categories identified and valued by the experts in the Delphi technique were not represented in the economic valuation literature in our database (*viz*: fodder, water bio-remediation, protection from salt intrusion, aesthetic value, pharmaceuticals and environmental risk indicator). While aesthetic value maybe evaluated to a certain degree based on the eco-tourism and recreation potential (e.g. [53]), the others may need appropriate indicators or new methods to be incorporated in the valuation framework because no other indicator covers them currently. The underlying ecological functions which lead the provisioning of these services may also need further attention. There was no significant correlation between ES ranks according to the two ranking approaches (Spearman's rank correlation coefficient r_s_  =  0.42, p = 0.23).

## Discussion

In this section, we focus on the implications for management and conservation of mangroves and future ES research. The recommendations broadly follow the CICES framework for ES classification and the current methods used to value ES.

### Provisioning services

The key provisioning services of mangroves identified were fisheries (important for subsistence, livelihood and commercial fisheries), wood and timber, honey, energy sources, fodder and pharmaceuticals. In developing countries where a large proportion of the rural poor population live on less than $1/day [54], these ES are crucial life support systems even though some of them might have a low monetary value (e.g. wood and timber) [27], [55]. Since all of these services are exhaustible in nature and the consumption or harvest by one individual reduces the available stock for the next individual, it is imperative to direct adequate management resources to safeguard these ES in the long term. As a first step, we recommend creating a baseline of the underlying ecological potential (stocks) of the mangrove forests in each site, based on ecological knowledge (scale dependent on the extent of the forest and the available management resources). As a second step, we recommend assessing the level of sustainable yield (flows) that can be supported by the chosen mangrove forest under consideration. Finally, we recommend the creation of conditions for recovery or effective restoration of mangrove forests in the areas where they existed in the past and creation of alternative resources (e.g. alternative energy and timber resources from managed plantations) for the consumption by local communities. In addition, investing resources in strengthening the cultural capital of the local communities may be useful for conservation. In developing countries, where non-monetized mechanisms have been in place for decades in managing common property resources, creating incentives for community based management of mangroves maybe more beneficial than payment for ES schemes as suggested by Gómez-Baggethun et al. [56] and Ostrom and Nagendra [57].

### Regulation and maintenance services

According to the CICES framework, half (eight out of sixteen) of the ES categories mentioned by the experts fall in the regulation and maintenance ES section, four in the top five ranking ES alone (considering the nursery element of the fisheries service). Out of these, only coastal protection has permeated adequately in economic valuation literature (nine estimates). These regulating ES do not have a direct market price in most cases and are estimated by contingent valuation methods (based on stated willingness to pay or willingness to accept a change). Therefore, it is harder to generate economic incentives for conservation of mangrove ecosystems in the short term. For example, unsustainable harvest of mangroves for provisioning services like timber or conversion of mangrove area to aquaculture ponds for short-term economic gain may seriously jeopardize their capability to provide regulating services like coastal protection in the long term. Recent studies indicate that the ecological bases for many of these ES are rapidly changing due to climate change and other impacts [58]. One of the many examples of severe consequences of mangrove destruction is the devastation caused by the recent typhoon Haiyan in November, 2013 in coastal areas of the Philippines where over half (approx. 51%) of mangroves have been destroyed in the last century alone [59]. Conservation and restoration of the ecological status is thereby urgently needed for continued existence of these ES. Strong cross-country policy measures and creation of mangrove protected areas may be useful in this regard particularly in countries where mangroves areas are shared between several nations such as Sunderbans. In addition, adequate valuation mechanisms (monetary and non-monetary) are needed for those ES categories mentioned by the experts but not represented in the literature. It is worth noting here that in several mangrove areas, the power to make decisions leading to either conservation/restoration of mangrove ecosystems may not lie on the local communities who depend on mangrove ES for their livelihood and subsistence. A recent study, [44] showed that degradation due to large scale development (e.g. building of highways, ports and harbours) is the biggest threat to mangroves globally. Often these large infrastructure development projects are initiated by bodies for whom the actual values (monetary and otherwise) of mangrove ES are non-existent.

### Cultural services

The two categories of cultural ES identified by the Delphi technique were ecotourism and recreation, and aesthetic services. While the economic valuations revealed that ecotourism has high economic potential, most of the estimates originated from the Caribbean islands and the Atlantic East Pacific (AEP) distribution of mangroves (also see [60], [61]). There could be two possible explanations; either ecotourism is infrequent in mangroves in the Indo West Pacific or there are fewer valuation exercises. If the latter is true then adequate economic valuations of the revenue generated by tourism, need to be done in the Indo West Pacific distribution of mangroves where the bulk of mangrove species and forests are located. One such example is the study conducted by Uddin et al. [53]. If the former is true and assuming that there is potential for sustainable tourism, then investing in the development of infrastructure for facilitating ecotourism in collaboration with local communities would be useful for the local economy. However, careful attention needs to be paid in choosing the appropriate incentives used for promoting tourism, i.e. the same principle of stock and flow applies here too for sustainable use of resources [13] or else there may be a collapse of the supply of ES. It should also be noted that there could be economic valuations in the grey literature or in other languages that were not covered in this study.

## Conclusions

This study complements the conventional monetary valuation of ES with non-economic valuation of ES by a range of mangrove experts who have been working in the field for over 20 years across the entire biogeographic range of mangroves. The deliberate primary focus on ecologists is both a bias and a scope of this study since ecosystem functioning is at the root of all the ES generation. Integrating the knowledge of ecologists who have prior experience in investigating ecosystem functioning of mangroves was our main aim. It should also be noted that some of the experts who participated and the authors who designed the questionnaire also had research experience on socio-economic assessments in mangroves [61]–[64]. This research adds to the existing literature on mangrove ES in an unconventional way and this approach can be easily replicated for other ecosystems (or later periods for the same questions). While expert consultation has been recommended by economists for estimating economic value transfer [16], this study approaches the issue of valuation by experts from an epistemological perspective rather than that of a merely technical nature for adjusting values [65] or identifying indicators[66]. It is thereby more in the lines of harnessing expert knowledge for progressing thought and improving the *status quo* as demonstrated by Wallington and Moore [67].

The lack of correlation between mangrove ES ranks based on expert based valuation (Delphi technique) and economic valuation, indicates that economic valuations may have missed out crucial ES and the scope of valuation of ES needs to be broadened. Even though both approaches show that mangrove ecosystems are particularly important for the provision of a range of ecosystem services, the relative importance of each of the ES categories differs markedly. Acknowledging this diversity, and developing methodological frameworks fostering value pluralism in ecosystem valuation research is key to maintaining the credibility of the ES valuation approach. Different valuation results might indeed lead to different trade-offs among ecosystem services [35] or other land uses. When decision-makers use ES valuation in guiding their plans for mangrove management and conservation [68], they need to be supported by critical analysis and a plurality of ES valuation methods to prevent mismanagement of these key tropical ecosystems. This certainly does not imply commodification of ES as claimed by [69] but rather the much needed acknowledgement of the ES which are often implicitly recognized in governance and policy issues [70].

Although the Delphi technique is a very useful method for expert knowledge elicitation, the use of the method might tend towards subjectivity, especially when dealing with complex systems as indicated in the study by Benitez-Capistros et al. [71]. This situation is linked to the inherent difficulty in determining who is a ‘knowledgeable respondent, i.e. an expert’ – a shortcoming we addressed by a transparent definition of the expert selection criteria (see material S1). Related to this problem are the difficulties of recruiting participants and avoiding dropouts in each round. Nevertheless, when conducted transparently, the Delphi technique is a rigorous method as it brings more objectivity and accuracy in the overall outcome [72]. In addition, in order to have stakeholders who cannot be easily reached through the Delphi technique as applied here, complementary methods like integral valuation may be necessary.

This paper goes further than the valuation alone, as it addresses the issues related to management options and future ES assessments for the continued sustainable use of mangrove ES in the long term. Further research on ES valuation in selected mangrove sites is needed to complement this global scale explorative research. Looking forward, an open question that remains to be answered is how the newly emphasized ES categories (lacking economic valuations) influence decision making in conservation, management and restoration of mangroves in the future.

## Supporting Information

Material S1 **Criteria for selection of experts for the mangrove Delphi survey.**
(DOCX)
