# Correction: Ecosystem Service Valuations of Mangrove Ecosystems to Inform Decision Making and Future Valuation Exercises

**DOI:** 10.1371/journal.pone.0111386

**Published:** 2014-10-13

**Authors:** The PLOS ONE Staff

The attribution for the original source of Figure 1 is missing. Please see the corrected Figure 1 caption here.

**Figure 1:**
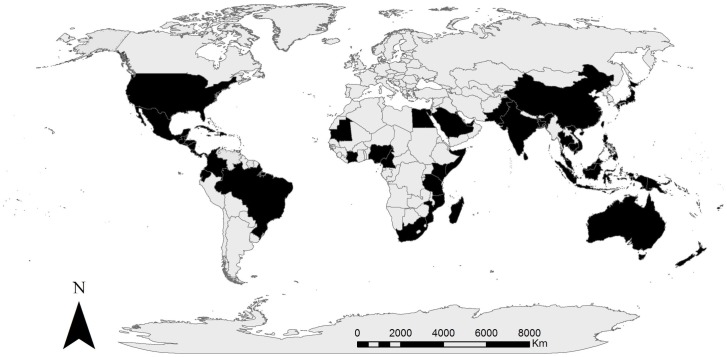
Map representing countries (coloured black) where the experts have conducted primary research on mangrove ecosystems. Adapted from DOI: 10.1002/ece3.1085.
