# Supplementary material for: Ecosystem Service Valuations of Mangrove Ecosystems to Inform Decision Making and Future Valuation Exercises
Source: PLoS One. 2014 Sep 22;9(9):e107706. doi: 10.1371/journal.pone.0107706 (PMC4171500; doi:10.1371/journal.pone.0107706)
Supplement: Material S1 — Criteria for selection of experts for the mangrove Delphi survey. (DOCX) [file pone.0107706.s001.docx]

**Supplementary Material for**

Ecosystem service valuations of mangrove ecosystems to inform decision making and future valuation exercises

Nibedita Mukherjee^1,2*^, William J. Sutherland^3^, Lynn Dicks^3^, Jean Hugé^4,1^, Nico Koedam^2,‡^ and Farid Dahdouh-Guebas^1,2,‡^

*^1^Laboratory of Systems Ecology and Resource Management, Université Libre de Bruxelles, CP 264/1, Avenue F.D. Roosevelt 50, B-1050 Brussels, Belgium,*

*^2^Laboratory of Plant Biology and Nature Management, Vrije Universiteit Brussel, Pleinlaan 2, B-1050 Brussels, Belgium,*

*^3^Conservation Science Group, Department of Zoology, University of Cambridge, Cambridge CB2 3EJ, England,*

*^4^Centre for Sustainable Development, Ghent University, Poel 16, 9000 Gent, Belgium*

*Corresponding author: Nibedita Mukherjee, Laboratory of Systems Ecology and Resource Management, Université Libre de Bruxelles, CP 264/1, Avenue F.D. Roosevelt 50, B-1050 Brussels, Belgium. E-mail: nibedita.41282@gmail.com

‡Co-last author

**This file contains:**

Criteria for selection of experts for the mangrove Delphi survey.

**DELPHI TECHNIQUE**

*Selection of experts*: The experts were chosen based four criteria

1. Number of peer-reviewed publications related to mangrove ecology: We searched for the keywords ‘mangrove’ and ‘ecology’ appearing together in ISI Web of Science® and identified the first 250 authors irrespective of co-authorship or author position. This list of 250 authors was then manually sorted.
2. Publications in high impact factor journals: We identified authors based on key research articles on mangroves in three high impact factor journals (Impact Factor>ten) - Science, Annual Review of Marine Science and Public Library of Science.
3. CREC project: We also selected mangrove experts working on an international European-Commission funded collaborative project on mangroves termed as Coastal Research network on Environmental Changes (2010-2014) in five continents (http://www.forst.tu-dresden.de/CREC/).
4. Managers and restoration biologists: We also invited mangrove managers and on-ground restoration biologists who were/are involved in mangrove research.

Based on the above short listing a total of 106 mangrove experts were finally selected. Care was taken to select experts outside our research group and the authors who supervised this research did not participate in this survey even though both FDG and NK are mangrove experts.
